# Transcriptome Modifications in the Porcine Intramuscular Adipocytes during Differentiation and Exogenous Stimulation with TNF-α and Serotonin

**DOI:** 10.3390/ijms21020638

**Published:** 2020-01-18

**Authors:** Asuka Tada, Md Aminul Islam, AKM Humayun Kober, Kohtaro Fukuyama, Michihiro Takagi, Manami Igata, Leonardo Albarracin, Wakako Ikeda-Ohtsubo, Kenji Miyazawa, Kazutoyo Yoda, Fang He, Hideki Takahashi, Julio Villena, Hisashi Aso, Haruki Kitazawa

**Affiliations:** 1 Food and Feed Immunology Group, Laboratory of Animal Products Chemistry, Graduate School of Agricultural Science, Tohoku University, Sendai 981-8555, Japan; a-tada@morinagamilk.co.jp (A.T.); aminul.vmed@bau.edu.bd (M.A.I.); fukuyama.k.mc0511@gmail.com (K.F.); takagimichihiro@gmail.com (M.T.); manamiigata@gmail.com (M.I.); lalbarracin@herrera.unt.edu.ar (L.A.); wakako.ohtsubo.a7@tohoku.ac.jp (W.I.-O.); jcvillena@cerela.org.ar (J.V.); 2 Livestock Immunology Unit, International Education and Research Center for Food Agricultural Immunology (CFAI), Graduate School of Agricultural Science, Tohoku University, Sendai 981-8555, Japan; 3 Department of Medicine, Faculty of Veterinary Science, Bangladesh Agricultural University, Mymensingh 2202, Bangladesh; 4 Laboratory of Immunobiotechnology, Reference Centre for Lactobacilli, (CERELA-CONICET), Tucuman 4000, Argentina; 5 Technical Research Laboratory, Takanashi Milk Products Co., Ltd., Yokohama Kanagawa, Yokohama 241-0023, Japan; ke-miyazawa@takanashi-milk.co.jp (K.M.); k-yoda@takanashi-milk.co.jp (K.Y.); KA-HOU@takanashi-milk.co.jp (F.H.); 6 Laboratory of Plant Pathology, Graduate School of Agricultural Science, Tohoku University, Sendai 981-8555, Japan; hideki.takahashi.d5@tohoku.ac.jp; 7 Plant Immunology Unit, International Education and Research Center for Food Agricultural Immunology (CFAI), Graduate School of Agricultural Science, Tohoku University, Sendai 981-8555, Japan; 8 Cell Biology Laboratory, Graduate School of Agricultural Science, Tohoku University, Sendai 981-8555, Japan

**Keywords:** porcine intramuscular preadipocyte (PIP), porcine mature adipocyte (pMA), serotonin, serotonin receptor, adipogenesis, TNF-α, transcriptome

## Abstract

Adipocytes are dynamic cells that have critical functions to maintain body energy homeostasis. Adipocyte physiology is affected by the adipogenic differentiation, cell program, as well as by the exogenous stimulation of biochemical factors, such as serotonin and TNF-α. In this work, we investigated the global transcriptome modifications when porcine intramuscular preadipocyte (PIP) was differentiated into porcine mature adipocyte (pMA). Moreover, we studied transcriptome changes in pMA after stimulation with serotonin or TNF-α by using a microarray approach. Transcriptome analysis revealed that the expression of 270, 261, and 249 genes were modified after differentiation, or after serotonin and TNF-α stimulation, respectively. Expression changes in *APP*, *HNF4A*, *ESR1*, *EGR1*, *SRC*, *HNF1A*, *FN1*, *ALB*, *STAT3*, *CBL*, *CEBPB*, *AR*, *FOS*, *CFTR*, *PAN2*, *PTPN6*, *VDR*, *PPARG*, *STAT5A* and *NCOA3* genes which are enriched in the ‘PPAR signaling’ and ‘insulin resistance’ pathways were found in adipocytes during the differentiation process. Dose-dependent serotonin stimulation resulted in a decreased fat accumulation in pMAs. Serotonin-induced differentially expressed genes in pMAs were found to be involved in the significant enrichment of ′GPCR ligand-binding′, ‘cell chemotaxis’, ‘blood coagulation and complement’, ‘metabolism of lipid and lipoproteins’, ‘regulation of lipid metabolism by *PPARA’*, and ‘lipid digestion, mobilization and transport’ pathways. TNF-α stimulation also resulted in transcriptome modifications linked with proinflammatory responses in the pMA of intramuscular origin. Our results provide a landscape of transcriptome modifications and their linked-biological pathways in response to adipogenesis, and exogenous stimulation of serotonin- and TNF-α to the pMA of intramuscular origin.

## 1. Introduction

Adipocytes are dynamic cells that have critical functions to maintain body energy homeostasis. The ability of adipocytes to store and mobilize energy is of fundamental physiological importance. Excess energy is mostly stored in the adipose tissue, and it is released as free fatty acids through a process that is regulated and inhibited by insulin [1]. The excessive accumulation of adipose tissue mass results in obesity, due to adipocyte hypertrophy without hyperplasia. In obese subjects, adipocytes are excessively enlarged and are associated with pathologic alterations, such as hypoxia, fibrosis, and inflammation with macrophage infiltration [2,3]. These alterations in the adipose tissue play a crucial role in the development of obesity-associated metabolic disorders, especially insulin resistance and type 2 diabetes mellitus [3]. Therefore, the mode of adipocyte expansion is critical for insulin sensitivity and overall metabolic health. The expansion of adipose depots can be driven either by the increase in adipocyte size called hypertrophy or by the formation of new adipocytes from precursors’ differentiation in the process of adipogenesis called hyperplasia [4]. Notably, adipocyte expansion through adipogenesis can offset the negative metabolic effects of obesity [4]. Adipocyte hyperplasia leads the activation of biological pathways that favor adipocyte differentiation, the formation of adipocytes from precursor cells, produces an increase in the number of physiologically functional adipocytes [4]. Preadipocytes are one kind of precursor cells from where adipocytes are likely generated through adipogenic differentiation. Therefore, elucidating the molecular mechanisms and the regulators of preadipocyte differentiation is necessary to enhance our understanding of the pathogenesis of obesity-associated metabolic diseases in pigs, as well as in humans.

Serotonin or 5-hydroxytryptamine (5-HT) is a biogenic monoamine that acts either as a neurotransmitter in the brain or as a hormone in the periphery. The peripheral serotonin is known to be involved many important biological functions, including vasoconstriction and heart rate in the cardiovascular system [5], appetite, homeostasis and gastrointestinal functions [6], inflammatory responses [7], and obesity [8,9]. Serotonin has shown to influence both lipid and glucose metabolism directly by suppressing lipolysis and glucose uptake in primary adipose cells in the rodent model [10]. Serotonin exerts its biochemical signals through its interaction with several classes of serotonin receptors distributed throughout the nervous system and peripheral organs like adipose tissue [6,11]. Though more than 90% of body’s serotonin is synthesized through hydroxylation of dietary L-tryptophan catalyzed by tryptophan hydroxylase 1 (TPH1) expressed in the enterochromaffin cells of the gastrointestinal tract [12], it was reported that adipocytes themselves are capable of producing serotonin [11]. Exogenous serotonin in like gut-derived serotonin can regulate the signaling through serotonin receptors (HTRs) in adipocytes [13]. We, therefore, checked the presence of serotonin receptors in the adipocytes followed by serotonin-induced gene expression changes in adipocytes originated from porcine intramuscular preadipocytes.

Hypertrophied and dysfunctional adipocytes in obese subjects lose their ability to minimize the adverse effect of a high-fat diet, and induce the secretion of proinflammatory cytokines, including tumor necrosis factor-alpha (TNF-α), which promotes to initiate local and systemic inflammatory responses [14]. TNF-α is synthesized by adipocytes and adipose tissue-infiltrated macrophages in obese hosts, acting as an endocrine and paracrine/autocrine mediator through interaction with type I and type II TNF-α receptors (TNFR I and II), both expressed in adipocytes [15]. It was reported that TNF-α positively contributes to maintaining the proinflammatory microenvironment through the activation of nuclear factor -kappa B (NF-κB) signaling [16], which leads significant changes in gene expression and thereby affecting adipocyte functionality [17]. Therefore, global transcriptome profiling of adipocytes with and without exogenous TNFα stimulation would provide an important clue to identifying the potential candidate genes associated with metabolic functions of adipocytes.

Previously we established a porcine preadipocyte (PIP) cell line from the *Musculus longissimus thoracis* of a Duroc pig which maintains a normal phenotype without transforming spontaneously even after long-term culture [18]. We used this cell line for the investigation of adipogenic differentiation, and we were able to establish a protocol to obtain functional mature adipocytes from PIP cells [18]. In a recent transcriptome study, we demonstrated that Toll-like receptors are activated in the porcine mature adipocytes (pMA), which were obtained from in vitro differentiation of PIP cells [19]. Mass progress has been made in adipocytes research in the last three decades; however, the molecular regulatory mechanisms underlying intramuscular adipocytes differentiation remains unclear. Though few studies have compared gene-expression patterns in undifferentiated and differentiated porcine intramuscular adipocytes [20,21,22,23], the influences of serotonin or TNF-α in the global transcriptome modifications of adipocytes are yet to be elucidated. In this study, we investigated the global gene expression changes during differentiation of PIP into pMA, and the influence of exogenous serotonin and TNF-α stimulation in the transcriptional modification of pMA.

## 2. Results

### 2.1. Transcriptome Signatures of PIP Cells Differentiation

**Figure 1:**
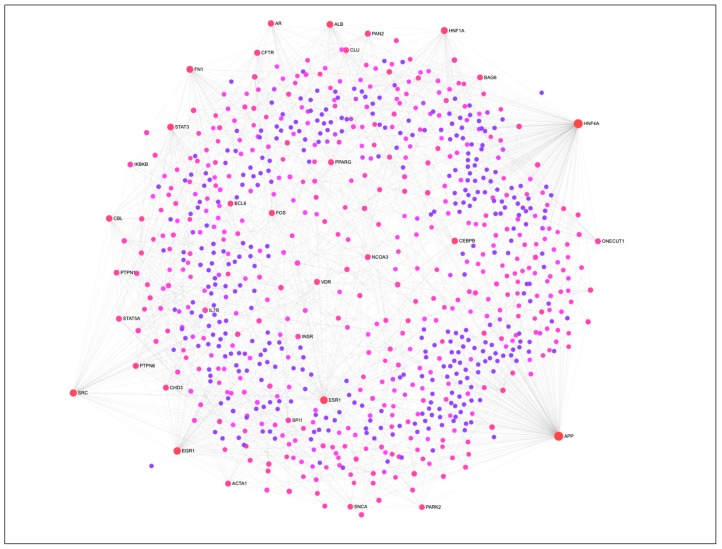
The protein-protein interaction (PPI) network of DEGs associated with adipogenesis in porcine intramuscular adipocyte. The PPI network was constructed by using NetworkAnalyst software incorporated with InnateDB interactome database. Circular nodes represent the differentially expressed genes, and edge represent the interaction. Circle diameter represents the degree centrality (number of connections it has to others), while the color intensity (from purple towards red) of a node represents the betweenness centrality (number of connections passing through this node) of the network.

The PIP cells were subjected to in vitro differentiation and maturation in vitro with specified growth media and maintained for four days. Then we investigated the global expression changes between PIP cells and pMA to explore the transcriptome signatures for the adipogenic differentiation. Microarray expression analysis identified a total of 270 differentially expressed genes (DEGs) in pMA when compared to PIP cells. The protein-protein interaction analysis was performed to detect the most potential regulatory Hub genes of the transcriptional network associated with adipogenesis (Figure 1). The top twenty potential network Hub genes included *APP*, *HNF4A*, *ESR1*, *EGR1*, *SRC*, *HNF1A*, *FN1*, *ALB*, *STAT3*, *CBL*, *CEBPB*, *AR*, *FOS*, *CFTR*, *PAN2*, *PTPN6*, *VDR*, *PPARG*, *STAT5A* and *NCOA3* (Figure 1).

**Figure 2:**
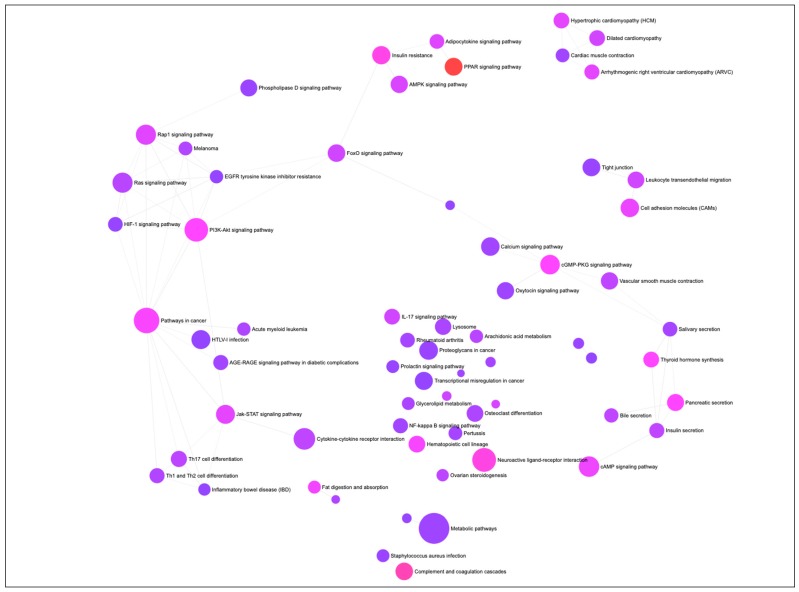
The gene-set network, showing KEGG (Kyoto Encyclopedia of Genes and Genomes) pathways enriched by the differentially expressed genes (DEGs) associated with adipogenesis in the porcine intramuscular adipocyte. Circular nodes represent the pathways and edge connected the biologically similar pathways. Node’s diameter represents the number of DEGs involved with the enrichment (the bigger size, the higher number of genes), and the color of the node reparent the adjusted *p*-value of significant enrichment (the more intense color from purple towards red, the higher significance).

A gene-set network was constructed to visualize the KEGG (Kyoto Encyclopedia of Genes and Genomes) pathways enriched by the DEGs associated with differentiation of PIP cells into pMA (Figure 2). The top ten significantly enriched KEGG pathways includes ‘PPAR signaling’, ‘Complement and coagulation cascades’, ‘Neuroactive ligand-receptor interaction’, ‘Insulin resistance’, ‘PI3K-Akt signaling’, ‘cGMP-PKG signaling’, ‘Thyroid hormone synthesis’, ‘Pancreatic secretion’, and ‘Fat digestion and absorption’ pathways (Figure 2).

### 2.2. Expression of Serotonin Receptor Proteins in PIP and pMA

**Figure 3:**
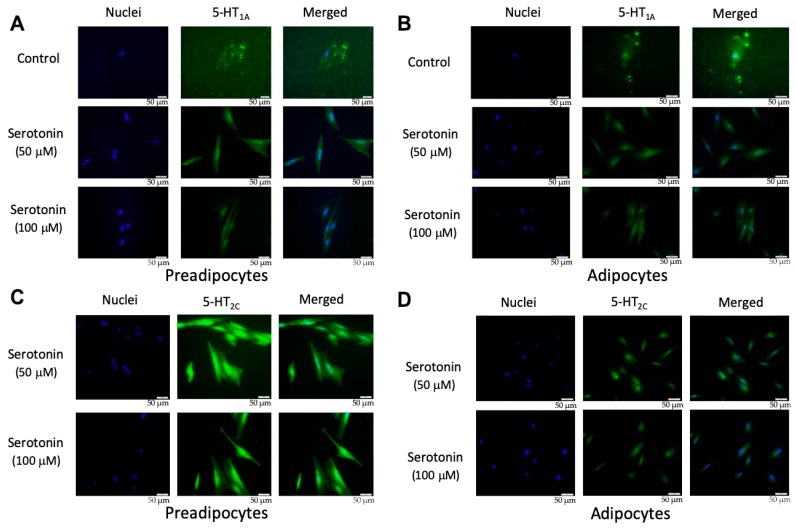
Expression of 5-HT_1A_ receptor in porcine intramuscular preadipocytes (PIP) (**A**) and in porcine mature adipocytes (pMA) of intramuscular origin (**B**); and the expression of the 5-HT_2C_ receptor in PIP (**C**) and in pMA (**D**). PIP cells and pMA cells were stimulated in vitro with serotonin (50 or 100 μM), and the protein level expression of 5-HT_1A_ and 5-HT_2C_ receptors was evaluated by immunofluorescent staining. Green color indicates the signal intensity of protein expression while blue color indicates the cellular nuclei. The upper most row of the images served as a control for the 5-HT_1A_ receptor in unstimulated cells (**A**, **B**), as well as for the isotype control of 5-HT_2C_ receptors (**C**, **D**).

There are seven serotonin receptor classes with a total of 14 different subtypes: 5-HT_1_ (5-HT_1A_, 5-HT_1B_, 5-HT_1D_, 5-HT_1E_, 5-HT_1F_), 5-HT_2_ (5-HT_2A,_ 5-HT_2B,_ 5-HT_2C_), 5-HT_3_, 5-HT_4_, 5-HT_5_(5-HT_5A,_ 5-HT_5B_), 5-HT_6,_ and 5-HT_7_, among which the activation of 5-HT_1A_ and 5-HT_2C_ receptors were reported to be involved in the regulation of adipocyte differentiation [24,25,26]. In order to confirm the presence of serotonin receptors, PIP cells and pMAs were stimulated with serotonin (50 or 100 μM), and the protein level expression of 5-HT_1A_ and 5-HT_2C_ receptors was evaluated by immunofluorescent staining. Results demonstrated that 5-HT_1A_ protein was expressed in PIP cells (Figure 3A) and in pMA (Figure 3B) after dose-dependent stimulation with exogenous serotonin. The 5-HT_2C_ protein was also expressed in PIP cells (Figure 3C) and in pMAs (Figure 3D) after the stimulation with exogenous serotonin.

**Figure 4:**
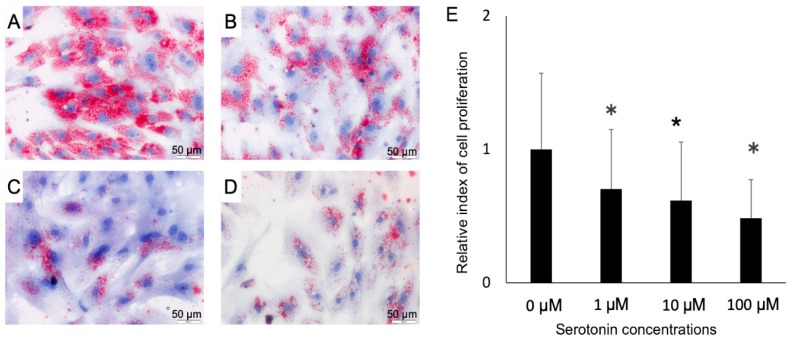
The Oil red O staining image is showing the serotonin-induced fat accumulation in- (**A**–**D**), and the rate of death/proliferation of adipocytes (**E**). Differentiated adipocytes were stimulated with 0 (control), 1, 10 or 100 μM and then fat accumulation was measured at four days post-stimulation by thin layer chromatography. **A**, control; **B**, 1 μM; **C**, 10 μM and **D**, 100 μM. Lipid droplets stained red color and cellular nuclei stained blue color (A–D). Results obtained from three independent trials are presented as mean ± standard deviation. * < 0.05, significant difference against the unstimulated control cells.

Once we confirmed the presence of serotonin receptors in the PIP and pMA, we explored the effect of serotonin stimulation on the fat accumulation in pMA. Oil red staining studies showed that serotonin stimulation resulted in a decreased accumulation of fat in the adipocytes in a dose-dependent manner (Figure 4A–D). There was significant cell death occurred following serotonin stimulation in dose-dependently (Figure 4E).

### 2.3. Transcriptome Modifications in pMA after Serotonin- or TNF-α -Stimulation

We investigated the patterns of gene expression changes in pMA after serotonin or TNF-α stimulation. The microarray data revealed that 261 and 249 genes were differentially expressed in pMAs after serotonin and TNF-α stimulation, respectively. A total of 181 DEGs were common between two stimulations, and differential expression of the shared genes followed the same direction. In both treatments, the number of upregulated genes were much higher than the down-regulated ones. DEGs were annotated and categorized into two major biological classes: Genes related to metabolic and endocrine function, and genes related to immune response function.

#### 2.3.1. Differential Expressions of Metabolic and Endocrine Genes in pMA after Serotonin- or TNF-α -Stimulation

**Figure 5:**
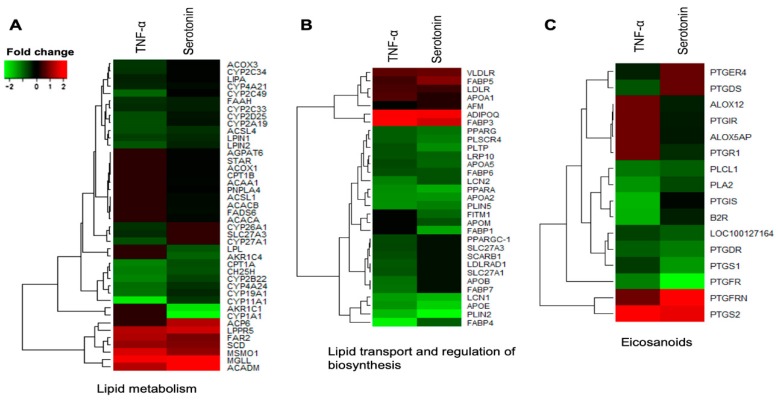
Genes associated with metabolic and endocrine functions which were differentially expressed in porcine mature adipocytes (pMA) of intramuscular origin after in vitro stimulation with serotonin (100 μM) and TNF-α (2.5 µg/mL) both for 12 h. Genes are presented as different functional groups: **A**. Lipid metabolism, **B**. Lipid transport and regulation of biosynthesis, and **C**. Eicosanoids. Green color indicates down-regulation and red color for up-regulation.

The DEGs associated with metabolic and endocrine function in adipocytes were clustered into three functional groups: Lipid metabolism, lipid transport and regulation of biosynthesis, and eicosanoids. Among the differentially expressed genes involved in lipid metabolism, *LPIN1, LPIN2, CYP11A1, CYP19A1, CYP2A19, CYP2B22, CYP2C33, CYP2C34, CYP2C49, CYP2D25, CYP4A21, CYP4A24, CH25H, ACOX3, FAAH, CPT1A, LIPA* and *ACSL4* were upregulated in pMAs after both serotonin and TNF-α stimulations, while *LPPR5, MSMO1, SCD, ACADM*, FAR2 and *MGLL* were down-regulated (Figure 5A). The expression of *ACACA, ACACB, FADS6, CYP1A1, AKR1C1, AKR1C4, STAR, CPT1B, ACOX1, ACAA1, LPL, PNPLA4, ACP6, AGPAT6* and *ACSL1* were upregulated in adipocytes after serotonin stimulation, but remain unchanged after TNF-α stimulation. On the other hand, expression of *SLC27A3*, *CYP26A1* and *CYP27A1*, were upregulated after TNF-α stimulation, but remain unchanged after serotonin stimulation (Figure 5A).

Among the differentially expressed genes linked to lipid transport and regulation of biosynthesis, *FABP4*, *FABP6*, *APOA2*, *APOA5*, *APOE*, *LRP10*, *PLSCR4*, *PLTP*, *LCN1*, *LCN2*, *PLIN5*, *PLIN2*, *PPARA* and *PPARG* were upregulated in adipocytes after both serotonin and TNF-α stimulations, while the expressions of *FABP3*, *FABP5*, *APOA1*, *LDLR*, *VLDLR* and *ADIPOQ* were down-regulated after both stimulations in adipocytes (Figure 5B). The expression of *FABP1*, *APOM* and *FITM1* were upregulated after serotonin stimulation, but remain unchanged after TNF-α stimulation. On the other hand, expression of *FABP7*, *APOB*, *SCARB1*, *SLC27A3*, *LDLRAD1*, *SLC27A1* and *PPARGC*-*1* were upregulated after TNF-α stimulation, but remain unchanged after serotonin stimulation (Figure 5B).

Different eicosanoids were differentially expressed in adipocytes after serotonin and TNF-α stimulation. Of which *B2R*, *PLA2*, *PLCL1*, *PTGDR*, *PTGS1*, *PTGIS*, and *PTGFR* were upregulated, while the expressions of only *PTGS2* was down-regulated after both stimulations in adipocytes (Figure 5C). The expression of *PTGR1*, *PTGIR*, *ALOX12* and *ALOX5AP* were upregulated, and expression of *PTGFRN* was down-regulated after serotonin stimulation, but their expression remains unchanged after TNF-α stimulation. On the other hand, expression of *PTGDS* and *PTGER4* were upregulated after TNF-α stimulation, but remain unchanged after serotonin stimulation (Figure 5C).

**Figure 6:**
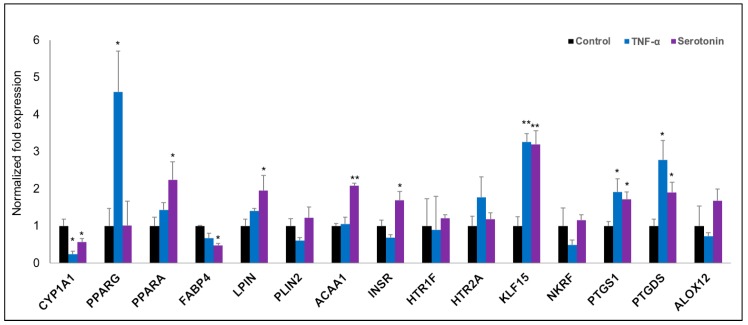
RT-qPCR validation of expression changes of selected metabolism-related genes in porcine mature adipocytes (pMA) of intramuscular origin. The pMA (2.5 × 10^4^ cm^2^) were stimulated with serotonin (100 μM) and TNF-α (2.5 µg/mL) both for 12 h. The normalized fold expression of each gene obtained from three independent experiments and represented in each bar as mean ±standard deviation. The asterisks (*, *p* < 0.05; **, *p* < 0.01) indicate statistically significant difference when compared with control.

In order to validate the microarray expression results, we quantified the mRNA expressions of fifteen selected differentially expressed genes biologically linked with metabolic and endocrine function by the RT-qPCR (Figure 6). The RT-qPCR-based expression changes of majority genes tested in pMA after stimulation with serotonin and TNF-α showed a similar trend as of microarray results. In particular, expressions of KLF15, PTGS1 and PTGDS were significantly upregulated; and CYP1A1 and FABP4 were significantly down-regulated in pMA after stimulation with serotonin and TNF-α as measured by RT-qPCR (Figure 6). Significant overexpression of PPARA, LPIN, ACAA1 and INSR genes were observed after serotonin stimulation, while overexpression of PPARG was observed after TNF-α stimulation to pMA (Figure 6).

#### 2.3.2. Differential Expressions of Immune Genes in pMA after Serotonin- or TNF-α -Stimulation

**Figure 7:**
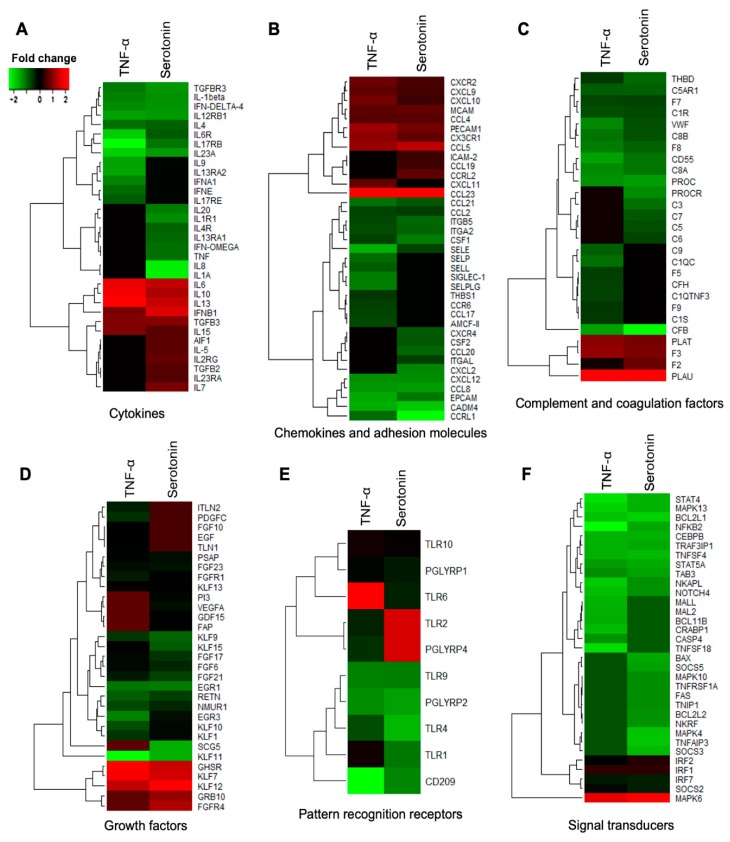
Immune response-related genes differentially expressed in porcine mature adipocytes (pMA) of intramuscular origin after in vitro stimulation with serotonin (100 μM) and TNF-α (2.5 µg/mL) both for 12 h. Genes are presented as different functional groups: **A**. Cytokines, **B**. Chemokines, **C**. Complement factors, **D**. Growth factors, **E**. Pattern recognition molecules, and **F**. Signal transduction molecules. Green color indicates down-regulation and red color for up-regulation.

The DEGs associated with immune response in adipocytes were further categorized into six subgroups: Cytokines, chemokine and adhesion molecules, complement and coagulation factors, growth factors, pattern recognition receptors, and signal transduction molecules. Among the cytokines, *IL1β*, *IL12RB1*, *IL17RB*, *IL23A*, *IL4*, *IL6R*, *IFN*-*δ4*, *TGFBR3* were upregulated in adipocytes after both TNF-α and serotonin stimulations, while the expressions of *IL10, IL13, IL15, IL6* and *IFNB1* were down-regulated after both stimulations (Figure 7A). The expression of *IL13RA1*, *IL1A*, *IL1R1*, *IL20*, *IL4R*, *TNF* and *IFN*-OMEGA were upregulated, and *IL23RA*, *IL2RG*, *IL5*, *IL7*, *AIF1* and *TGFB2* were downregulated in adipocytes after serotonin stimulation, but remain unchanged after TNF-α stimulation. On the other hand, expression of *IL13RA2*, *IL15*, *IL17RE*, *IL9*, *IFNA1, IFNE* and *IL13RA2* were upregulated after TNF-α stimulation, but remain unchanged after serotonin stimulation (Figure 7A).

Among the chemokines and adhesion molecules differentially expressed in adipocytes, *CCL2*, *CCL21*, *CCL8*, *CCRL1*, *CSF1*, *CXCL12*, *CADM4*, *EPCAM*, *ITGA2*, *ITGB5* and *SELE* were upregulated in adipocytes after both TNF-α and serotonin stimulations, while the expressions of *CX3CR1*, *CXCL10*, *CXCL9*, *CXCR2*, *CCL23*, *CCL4*, *CCL5*, *MCAM* and *PECAM1* were down-regulated after both stimulations (Figure 7B). The expression of *CCL20*, *CSF2*, *CXCL2*, *CXCR4* and *ITGAL* were upregulated, and *CCL19*, *CCRL2* and *ICAM*-2 were downregulated in adipocytes after serotonin stimulation, but remain unchanged after TNF-α stimulation. On the other hand, expression of *CCL17*, *CCR6*, *SELLL*, *SELP*, *SELPLG*, *SIGLEC-1* and *THBS1* were upregulated after TNF-α stimulation, but remain unchanged after serotonin stimulation (Figure 7B).

Several complement factors and coagulation factors were differentially expressed in adipocytes, among them *C1R*, *C5AR1*, *C8A*, *C8B*, *CFB*, *CD55*, *F7*, *F8*, *PROC*, *THBD* and *VWF* were upregulated while the expressions of *F3*, *PLAT* and *PLAU* were down-regulated after both serotonin and TNF-α stimulations (Figure 7C). The expression of *C3*, *C5*, *C6*, *C7* and *PROCR* were upregulated, and *C5AR1* and *F2* were downregulated in adipocytes after serotonin stimulation, but remain unchanged after TNF-α stimulation. On the other hand, *C1QC*, *C1QTNF3*, *C1S*, *C9*, *CFH*, *F5*, and *F9* expression were upregulated after TNF-α stimulation, but remain unchanged after serotonin stimulation (Figure 7C).

Of the growth differentially expressed after serotonin and TNF-α stimulations, *EGR1*, *EGR3*, *FGF21*, *FGF6*, *FGFR1*, *FGF17*, *FGF23*, *KLF1*, *KLF10*, *KLF11*, *KLF13*, *KLF15*, *KLF9*, *NMUR1*, *PSAP* and *RETN* were upregulated in adipocytes after both serotonin and TNF-α stimulations, while the expressions of *KLF12*, *KLF7* and *GHSR* were down-regulated after both stimulations in adipocytes (Figure 7D). The expression of *FAP*, *GDF15*, *SCG5, VEGFA* and *PI3* were upregulated, and *FGFR4* and *GRB10* were downregulated after serotonin stimulation, but their expressions remain unchanged after TNF-α stimulation. On the other hand, expression of *EGF*, *FGF10*, *PDGFC*, *TLN1* and *ITLN2* were upregulated after TNF-α stimulation, but remain unchanged after serotonin stimulation (Figure 7D).

Pattern recognition receptors, in particular, the *PGLYRP1*, *PGLYRP2*, *TLR1*, *TLR10*, *TLR4*, *TLR9* and *CD209* were upregulated in adipocytes after both TNF-α and serotonin stimulations (Figure 7E). Expression of *PGLYRP4* and *TLR2* were upregulated after TNF-α stimulation, but remained unchanged after serotonin stimulation, while *TLR6* was upregulated after serotonin stimulation, but remain unchanged after TNF-α stimulation (Figure 7E).

Among the signal transduction molecules differentially expressed in adipocytes, *TAB3*, *TNFSF4*, *TRAF3IP1*, *MAPK13*, *NOTCH4*, *STAT4*, *STAT5A*, *NFKB2*, *NKAPL*, *BCL2L1* and *CEBPB* were upregulated, while *MAPK6*, *SOCS2, IRF1, IRF2* and *IRF7* were downregulated after both TNF-α and serotonin stimulations (Figure 7F). The expression of *TNFAIP3*, *TNFRSF1A*, *TNIP1*, *MAPK10*, *MAPK4*, *NKRF*, *SOCS3*, *SOCS5*, *BCL2L2* and *FAS* were upregulated in adipocytes after serotonin stimulation, but remained unchanged after TNF-α stimulation. On the other hand, *TNFSF18*, *MAL2*, *MALL*, *BCL11B*, *CASP4* and *CRABP1* expression were upregulated in adipocytes after TNF-α stimulation, but remain unchanged after serotonin stimulation (Figure 7F).

**Figure 8:**
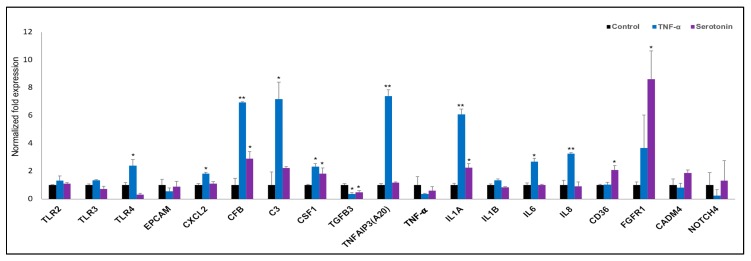
RT-qPCR validation of expression changes of selected immune response-related genes in porcine mature adipocytes (pMA) of intramuscular origin. The pMA (2.5 × 10^4^ cm^2^) were stimulated with serotonin (100 μM) and TNF-α (2.5 µg/mL) both for 12 h. The normalized fold expression of each gene obtained from three independent experiments and represented in each bar as mean ± standard deviation. The asterisks (*, *p* < 0.05; **, *p* < 0.01) indicate statistically significant difference when compared with control.

Finally, we confirmed the expression of nineteen differentially expressed genes in adipocytes after stimulation serotonin and TNF-α as measured by RT-qPCR (Figure 8). TLR4, CXCL2, C3, TNFAIP3, IL6 and IL8 were significantly upregulated after TNF-α stimulation, while the expression of only CD36 was upregulated after serotonin stimulation to pMA (Figure 8). In addition, significant overexpressions of CFB, CSF1 and IL1A, under expression of TGFB3 was observed in pMA after stimulation of both serotonin and TNF-α stimulation (Figure 8).

### 2.4. Pathways Activation by Serotonin- and TNF-α-Induced Transcriptome Alterations

**Figure 9:**
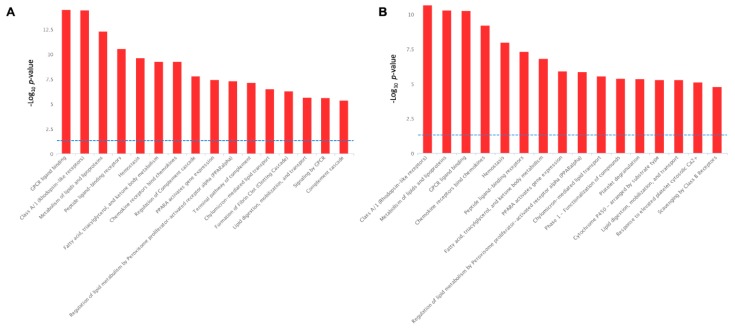
Biological pathways significantly enrichment by the serotonin-induced (**A**) and TNF-α-induced (**B**) differentially expressed genes in porcine mature adipocytes (pMA) of intramuscular origin. The -Log_10_ *p*-value of 1.3 was considered as the significant threshold as indicated by the blue dotted line.

For biological interpretation of the dataset, we performed the pathway over-representation analysis of DEGs using the REACTOME pathway database incorporated into InnateDB pathway analysis software. The serotonin-induced DEGs revealed the significant over-representation of several biological pathways, including ‘GPCR ligand binding’, ‘Signaling by GPCR’, ‘Metabolism of lipids and lipoproteins’, ‘Peptide ligand-binding receptors’, ‘Fatty acid, triacylglycerol, and ketone body metabolism’, ‘Regulation of lipid metabolism by PPARA’, ‘Lipid digestion, mobilization, and transport’, ‘Chylomicron mediated lipid transport’, ‘Homeostasis’, ‘Class A/1 (Rhodopsin-like receptors’, ‘Chemokine receptors bind chemokines’, and ‘Regulation of complement cascade’ (Figure 9A).

On the other hand, the TNFα-induced DEGs in adipocytes have shared many metabolic pathways with varying degree of significance, with those of serotonin-induced alterations. For instance, ‘Class A/1 (Rhodopsin-like receptors’, ‘Metabolism of lipids and lipoproteins’, ‘GPCR ligand binding’, ‘Peptide ligand-binding receptors’, ‘Fatty acid, triacylglycerol, and ketone body metabolism’, ‘Regulation of lipid metabolism by PPARA’, ‘Lipid digestion, mobilization, and transport’, ‘Chylomicron mediated lipid transport’, ‘Homeostasis’, ‘Chemokine receptors bind chemokines’, and ‘Regulation of complement cascade’ all these pathways are activated both TNFα stimulation (Figure 9B) and in serotonin stimulation (Figure 9A). In addition, unlike the serotonin stimulation, TNFα stimulation activates the biological pathways, such as ‘Platelet degranulation’, ‘Phase 1 functionalization of compound’, ‘Cytochrome P450 arranged by substrate type’, ‘Response to elevated platelet cytosolic Ca^2+^’, and ‘Scavenging by class B receptors’ pathways (Figure 9B).

## 3. Discussion

Central and peripheral serotonin systems are functionally separated as serotonin cannot pass the blood-brain barrier. Serotonin functions either as a central neurotransmitter or a circulatory hormone, and play distinct physiological roles depending on binding with specific serotonin receptors (HTRs) [27,28]. For instance, central serotonin increases adipose tissue thermogenesis, while peripheral serotonin inhibits it [27]. The biological effects of serotonin are mediated through interactions of 14 different subtypes of receptor proteins, of which 13 are G-protein coupled receptors, and only HTR3 (5-HT_3_) is a ligand-gated ion channel [24]. In this study, we confirmed the expressions of 5-HT_1A_ and 5-HT_2C_ proteins in pMA as compared to that of PIP cells. Both 5-HT_1A_ and 5-HT_2C_ receptors have been implicated in the regulation of body weight or obesity [27], indicating their expression and functionality in adipocytes [12]. Central serotonin functions as an anorexigenic neurotransmitter through activating the 5-HT_2C_ receptor [29], while peripheral serotonin signals through other HTRs in adipocytes [14]. After synthesizing from the enterochromaffin cells lining the gut, serotonin is carried by the platelets and mast-cells to the site of inflammation [30], suggest that exogenous serotonin could influence the adipocytes function in vitro. A previous study has pointed out the peripheral action of serotonin in regulating systemic energy homeostasis, and of serotonin receptors 5-HT_2A_ and 5-HT_3_ receptors in mediating the adipogenic effect of serotonin in adipose tissue [26]. It has also been reported that adipose deletion of 5-HT_2B_ results in blunted fasting-inducing lipolysis and nearly complete ablation of serotonin-induced lipolysis [14]. Another study reported that treatment with 5-HT_2A_ receptor agonist in the 3T3-L1 adipocytes resulted in blockage of lipid accumulation [10]. Therefore, the serotonergic effect in the adipocytes may differ among which serotonin-receptors it bonded with, and between the developmental stages of adipocytes.

Differentiation of preadipocytes into mature adipocyte is one of the most important biological processes that regulate adipose tissue development and adipocyte physiology [31]. The differentiation progresses through a cascade of molecular genetic events involving sequential activation of a set of transcription factors [32]. To explore the transcriptome signatures of adipogenic differentiation, we identified the Hub genes of the transcriptional network associated with differentiation. The differentiation-induced altered transcriptomes resulted in most significant enrichment of PPAR signaling pathway. The mRNAs of *PPARA, PPARG* and *CEBPB* were overexpressed in adipocytes compared to preadipocytes, and *PPARG* and *CEBPB* were found to be as potential Hubs gene of the PPI network. Previous studies have reported *PPARG* and *CEBPA* as potential markers genes associated with differentiation during preadipocyte differentiation into fully mature adipocytes [33]. *PPARG* is found to play a directorial role in the adipogenic hierarchy of transcription factors, while *CEBPA* promotes specific aspects of the adiposity phenotype [34]. With the expression of these genes, lipid droplets begin to appear in the cytoplasm and over time coalesce into fewer major droplets in the cells [35]. In a recent study, primary preadipocyte of Wujin pigs showed higher expression of *PPARG* and *CEBPA* genes during one to three hours of differentiation [23]. These results support the involvement of ‘PPAR signaling pathway’ in the preadipocytes differentiation of pigs.

Dose-dependent serotonin stimulation resulted in a decreased fat accumulation in the adipocytes, as well as decreased adipocyte proliferation. This results in accord with the previous findings [10] who reported that in vitro treatment with 5-HT_2A_ receptor agonist resulted in inhibition of lipid accumulation in 3T3-L1 adipocytes [10]. On the contrary, pharmacological inhibition of serotonin synthesis protects mice from high fat diet-induced obesity through decreasing adipose tissue lipogenesis, increasing browning in subcutaneous white adipose tissue, and increasing thermogenesis in brown adipose tissue [9]. Serotonin appears to be locally synthesized as an autocrine factor in adipocytes, since adipocyte-specific deletion of Thp1 results in a similar phenotype as the systemic loss of Thp1 [10]. Collective evidence suggests that peripheral serotonin has opposite metabolic function depending on the nature of food intake, and physiological state [27]. During fasting state, serotonin stimulates white adipose tissue lipolysis, whereas following high-fat diet, serotonin enhances the adipose tissue lipogenesis, as well as inhibit adaptive thermogenesis [14,27]. The complexity of peripheral serotonin signaling also attributed by the existence of multiple production sites, multiple secretory nature as of auto-, para- and endocrine, and a high number of serotonin receptors [25]. Therefore, influences of in vitro serotonin stimulation to adipocytes on the state of obesity requires further confirmation using in vivo experiments.

Serotonin-induced DEGs in pMAs participated in the significant enrichment of GPCR ligand-binding, Rhodopsin-like (Class A/1) receptors, and peptide ligand-binding receptor pathways. G-coupled protein receptors (GPCRs) are a receptor super family having more than 800 members, encompassing the largest class of ligands and drug targets. GPCRs can be classified in five different families: Rhodopsin (class A/1), secretin (class B1), adhesion (class B2), glutamate (class C), and frizzled/taste2 (class F) [36]. Rhodopsin-like receptors are the largest class among GPCR families, where 5-HTRs are included along with another hormone, light and neurotransmitter receptors [37]. Though GPCR has a high diversity in ligand-binding mechanisms, signaling from 5-HTR is similar to standard GPCRs signaling that mostly involves signal transduction via heterodimeric G-proteins. Except one, all serotonin receptors are also included in the GPCR family. Thus, enrichment of GPCR ligand-binding may explain the functional roles of serotonin stimulation in the adipocytes. GPCR function is associated with cell sensing of external factors, including light, metals, neurotransmitters, biogenic amines, fatty acids, amino acids, peptide, proteins, steroids and other lipids. GPCRs have been involved in many physiological and pathological conditions, including pain, asthma, cancer, cardiovascular diseases, gastrointestinal diseases, neurological diseases [38]. However, further computational approaches could attempt for more fine-tuning of serotonin functionality through GPCR ligand binding in adipocytes.

Serotonin stimulation resulted in overexpression of several immune genes involving cell chemotaxis, blood coagulation, and complement pathways. Once released from peripheral non-neuronal tissue, serotonin transport to the site of inflammation through mast cells and platelets and exerts its biological action by binding to serotonin receptors. It has been reported that 5-HT receptors signaling influences the regulation of lymphocyte B cell proliferation [39]. Serotonin has been shown to influence a number of immunological processes, and can lead to both increases and decreases in proinflammatory cytokines [40]. Peripheral serotonin signaling promotes the recruitment of neutrophils to the site of inflammation through the chemotactic process [41]. Studies using Tph1-deficient mice showed that serotonin exacerbates the development of inflammatory disease (e.g., colitis) through promoting secretion of proinflammatory cytokines [42]. Therefore, we postulate that serotonin signaling is involved in immunomodulation of the pMA of intramuscular origin.

Serotonin-induced DEGs were predicted to be involved in the regulation of several pathways, including the metabolism of lipid and lipoproteins; fatty acids, triacylglycerol and ketone body metabolism; regulation of lipid metabolism by *PPARA*; and lipid digestion, mobilization and transport pathways. Accumulating evidence suggests that peripheral serotonin acts as an endocrine factor to regulate metabolic function in multiple tissues, including adipose tissue [35]. The peripheral serotonin is known to exert effects through specific serotonin receptors to promote metabolic function, such as nutrient absorption and storage, while inhibiting thermogenesis [28]. Both gut-derived circulating serotonin and adipocyte-derived serotonin has implicated in the metabolic function of adipose tissue [27]. It has also reported that the administration of serotonin increases the levels of circulating non-esterified fatty acid and glycerol; and fasting increases the circulating serotonin level [14]. Altogether, peripheral serotonin signaling is highly likely to be involved in the regulation of the metabolic activity of adipocytes.

TNF-α is a multi-functional cytokine that can regulate many biological processes, such as immune function, and adipocyte metabolism [43]. TNF-α-induced transcriptional modification follows a similar trend, and alteration of many genes shared between both serotonin- and TNF-α stimulations. Like serotonin, TNF-α stimulation also activated the GPCR, peptide-ligand binding pathway and cytokine receptors binding pathways. Proinflammatory adipokine profile is associated with obesity and is thought to promote insulin resistance [44], while adiponectin and other anti-inflammatory adipokines are reduced in obesity and therefore might contribute to the maintenance of insulin sensitivity [45]. TNF-α stimulation directly disrupts signaling downstream of the insulin receptor, primarily via inflammatory kinase, such as an inhibitor of NF-kB kinase unit beta (IKK-β) [46], cJun NH (2)-terminal kinase (JNK) [47], and extracellular-signal-regulated protein kinase (ERK) [48]. Transcriptionally, TNF-α signaling through NF-kB kinase down-regulate the expression of *PPARG*. TNFα-induced NF-kB further regulate the transcription of other insulin response genes, which decrease insulin sensitivity in the adipocytes [17].

In addition, TNF-α-induced differentially expressed genes are involved with regulation of the metabolism of lipid, lipoprotein, fatty acids, and ketone body. Many previous studies support the involvement of TNF-α in lipid metabolism. TNF-α is expressed in adipose tissue, and its mRNA and protein levels are significantly increased in adipose tissue of obese animals [49]. A persistent increase of circulating levels of TNF-α occurring during obesity or aging has an important role in pathogenesis of systemic insulin resistance [17]. Moreover, TNF-α signaling can trigger adipocyte lipolysis by increasing expression of the enzyme-like hormone sensitivity lipase and adipose triglycerides lipase that breakdown triglycerides into free fatty acids (FFAs) and promotes their release from the adipocytes [50]. Over time, these FAAs can accumulate in distal organs, such as the liver and muscle resulting metabolic dysfunction, promoting a vicious cycle during obesity [51]. Taken together, TNF-α might be involved in regulating the immunometabolism of pMA of intramuscular origin.

## 4. Materials and Methods

### 4.1. Cell Line and Culture Conditions

Porcine intramuscular preadipocyte (PIP) cells, which are derived from marbling muscle tissue of the musculus longissimus thoracis from female Duroc pig [18], were maintained in Dulbecco’s modified Eagle medium (DMEM, Gibco, Paisley, Scotland, UK) supplemented with 10% fetal calf serum (FCS), 100 mg/mL penicillin, and 100 U/mL streptomycin as a growth medium. PIP cells were plated at a density of 2.5 × 10^4^ cm^2^ in 6-well cell culture plates (BD Falcon, Tokyo, Japan) and incubated at 37 °C in a humidified atmosphere of 5% CO_2_. The medium was changed every day.

### 4.2. Differentiation of PIP into Mature Adipocytes

The 4-day post-confluent Porcine intramuscular preadipocyte (PIP) cells were fed with differentiation medium for another four more days to yield the differentiated adipocyte. The differentiation medium was prepared by DMEM with 10% FBS, 50 ng/mL swine insulin (Sigma-Aldrich, Tokyo, Japan), 0.25 μM dexamethasone (Sigma-Aldrich, Tokyo, Japan), 2 mM octanoate (Wako, Osaka, Japan), 200 μM oleate (Ardorich, Milwaukee, WI, USA), 100 U/mL penicillin, and 100 μg/mL streptomycin.

### 4.3. Proliferation Assay and Oil Red O Staining of Adipocytes

For optimizing the dose of serotonin, we first stimulated the pMA cells (2.5 × 10^4^ cm^2^) with serial dilution of serotonin and evaluated the cell proliferation by cell counting, then 0.5% Oil red O (Sigma-Aldrich, Tokyo, Japan) in isopropanol was added to the cells for 5 min to visualize lipid droplets stained red. The cytosolic triglyceride content was analyzed using LabAssay™ triglyceride kit (FUJIFILM Wako Chemicals USA, Corp., Osaka, Japan) according to the manufacturer’s protocol.

### 4.4. Immunofluorescent Staining

The serotonin receptor agonist-treated and untreated cells were washed for 24 h with 1% gum arabic in 0.1 M phosphate buffer containing 8% sucrose and for additional 24 h with the same solution containing 16% sucrose. The cells were incubated with Blocking One Histo (Nacalai Tespue Inc., Kyoto, Japan) to block non-specific binding sites. After removal of the blocking solution, sections were incubated for 16 h at 4 °C in a humidified chamber with 1:1000 anti-5-HT_1A_ polyclonal antibody (Bios Antibodies Inc, Tokyo, Japan) or 1:1000 anti- 5-HT_2C_ polyclonal antibody (Bios Antibodies Inc, Tokyo, Japan). After washing with PBS, sections were incubated for 60 min with 1:1000 Alexa 488-conjugated goat anti-rabbit IgG F(ab’)2 (ThemoFisher Scientific, Yokohama, Japan). Double immunostaining for pan cytokeratin, and either 5-HT_1A_ or 5-HT_2C_ was also performed using 1:2000 anti-pan cytokeratin monoclonal antibody (clone C-11; Sigma-Aldrich, St. Louis, MO, USA) followed by 1:1000 Alexa 546-conjugated goat anti-mouse IgG F(ab’)2 (ThemoFisher Scientific, Yokohama, Japan). Then, samples were washed three times with PBS and stained with DAPI (Dojindo Laboratories, Kumamoto, Japan) to detect nuclei. Finally, the tissue sections were washed three times with PBS, mounted in ProLong Gold (ThemoFisher Scientific, Yokohama, Japan), and observed under an FSX100 microscope (Olympus, Tokyo, Japan). Control experiments were performed by omitting primary antibodies.

### 4.5. Stimulation of Adipocytes with Serotonin and TNF-α

The differentiated pMA cells were cultured at a density of 2.5 × 10^4^ cm^2^ in 6 well or 12 well plates (BD Falcon, Tokyo, Japan). The 4-day post-confluent pMA cells were stimulated with serotonin (100 μM) and TNF-α (2.5 µg/mL) both for 12 h.

### 4.6. RNA Isolation and Quality Control

Total RNA was isolated from the undifferentiated PIP, differentiated pMA without stimulation, differentiated pMA after serotonin stimulation, and differentiated pMA after TNF-α stimulation, using PureLink RNA Mini Kit (Life Technology Inc., Gathersburg, MD, USA) along with on-column DNase treatment. RNA integrity, quality and quantity were evaluated with microcapillary electrophoresis (2100 Bioanalyzer, Agilent Technologies, Santa Clara, CA, USA) using the RNA 6000 Nano kit. Only samples with an RNA integrity number (RIN) of greater than 8 were used for further analysis.

### 4.7. RNA Labeling and Microarray Hybridization

Two-hundred nanograms of total RNA pooled from each group was used to generate cDNA and Cy3-labeled cRNA using the Low Input Quick-Amp Labeling Kit (Agilent Technologies, Santa Clara, CA, USA), according to the manufacturer’s instructions. The labeled cRNA was photometrically examined to determine the quantity and dye-incorporation ratio using an Ultraspec 2000, and it was hybridized to a Mouse Gene Expression 4 × 44K v2 Microarray (Agilent Technologies). The array was scanned with GenePix 6000B (Molecular Devices, Sunnyvale, CA, USA), and the obtained image was processed using GenePix Pro 6.0 Software (Molecular Devices). Features were manually examined, and poor-quality spot, were flagged and filtered out.

The microarray hybridization was performed with a two-color with Porcine (V2) Gene Expression Microarray 4 × 44K oligonucleotide slide (Agilent, USA), containing 43,803 probes for the identification of known genes of the porcine transcriptome. The microarray experiment was conducted at Hokkaido System Science Co., according to the Two-Color Microarray-Based Gene Expression Analysis protocol v6.7 (Agilent Technologies, USA). For each sample, 200 ng of total RNA was converted into cDNA by reverse transcription. The cDNA was subsequently transcribed into cRNA and labeled with two fluorochrome dyes: Cyanine 3 (Cy3) and cyanine 5 (Cy5). About 1.65 μg of labeled cRNA was mixed with hybridization buffer and hybridized on microarray slide (4 samples in each slide) for 17 h at 65 °C with constant rotation. After hybridization, microarrays were cleaned with Gene Expression wash buffer and scanned using High-Resolution Microarray Scanner (Agilent Technologies, USA). The Feature Extraction software (v10.7.3.1, Agilent Technologies, USA) was used for detailed analysis of scanned images, including filtering the outlier spots, background subtraction from features and dye normalization. The spot intensity data for the individual sample were extracted for statistical analysis.

### 4.8. Statistical Analysis of Microarray Data

The normalization and expression analysis of the microarray data were performed with GeneSpring GX software (v13.1, Agilent Technologies, USA). Normalized expression results were summarized as log_2_ transformed scale for each transcript. Statistical analysis was performed with the linear analysis of microarray technique from ‘limma’ package [52]. Genes with significant changes in transcript abundance were selected on the basis of two criteria: A t-test *p*-value of less than 0.05, which was considered statistically significant; and a cutoff in transcript abundance of at least 2-fold.

### 4.9. Pathway Analysis

For biological interpretation of differential gene expressions, pathway enrichment analyses were performed using the InnateDB online tool v5.4 [53] using REACTOME pathway database. The ensembl gene ID was uploaded to the InnateDB web portal and performed the biological pathway analysis. Then over-representation analysis was performed using hypergeometric algorithm with Benjamini-Hochberg (B-H) multiple test correction method. Finally, the overrepresented pathways were filtered by a significant threshold of log10 *p*-value as 1.3.

### 4.10. Network Analysis

In order to visualize the differentiation-induced transcriptional network, as well as to identify the regulatory genes, the protein-protein interaction network analysis was performed using NetworkAnalyst online tool [54]. Human orthologous gene symbol of the common DEGs from all three stimulation were uploaded into the NetworkAnalyst to construct the interaction network based on Walktrap algorithm taking only direct interaction of seed genes. The network was depicted as nodes (circles representing genes) connected by edges (lines representing direct molecular interactions). Two topological measures, such as degree (number of connections to the other nodes) and betweenness (number of shortest paths going through the nodes) centrality were taken into account for detecting highly interconnected genes (Hubs) of the network. Nodes having a higher degree and betweenness were considered as potentially important Hubs in the cellular signal trafficking. In addition, gene-set network for the KEGG pathways enriched by differentiation-induced DEGs was also constructed.

### 4.11. Validation of Microarray Expression by RT-qPCR

Two-step real-time quantitative PCR (RT-qPCR) was performed to validate the microarray results by quantifying the expression of 29 selected mRNAs in PIP cells. Total RNA was isolated from each sample using TRIzol reagent (Invitrogen, Carlsbad, CA, USA). In order to remove the genomic DNA, the samples were treated with gDNA Wipeout Buffer (Qiagen, Tokyo, Japan). All cDNAs were synthesized using a Quantitect reverse transcription (RT) kit (Qiagen, Tokyo, Japan) according to the manufacturer’s recommendations. Real-time quantitative PCR was carried out using a 7300 real-time PCR system (Applied Biosystems, Warrington, UK). The RT-qPCR was performed using a 7300 real-time PCR system (Applied Biosystems, Warrington, UK) and the TaqMan^®^ gene expression assay kit (Life Technologies), TaqMan^®^ Universal Master Mix II, with UNG (Applied Biosystems, Warrington, UK). The PCR cycling conditions were 2 min at 50 °C, followed by 10 min at 95 °C, and then 40 cycles of 15 s at 95 °C, 1 min at 60 °C. The reaction mixtures contained 2.5 μL of sample cDNA, 1 μL gene expression assay and 10 μL TaqMan^®^ Universal Master Mix II, with UNG, and 6.5 μL distilled water. Expression of β-actin in each sample was assessed, and the β-actin data were used as an internal control to normalize differences between samples and to calculate relative expression levels. According to the minimum information for publication of quantitative real-time PCR experiments guidelines, β-actin was used as a housekeeping gene because of its high stability across various porcine tissues [55]. The relative index was calculated as the ratio of target mRNA expression to β-actin. Sequences of the mRNA primers used are shown in Supplementary information Table S1.

### 4.12. Statistical Analysis of RT-qPCR Data

The raw data were transferred from the mean CT values of replicated samples to copy number according to the established standard curve. The raw data were log transformed, followed by checked the normality by Kolmogorov-Smirnov test and convergence by club′s rejection test. Statistical analyses were performed using GLM and REG procedures available in the SAS computer program [56]. Comparisons between mean values were carried out using one-way ANOVA and Fisher’s least significant difference test. The statistical comparison of the relative index of cell proliferation between the treated and untreated group was performed by using a non-parametric chi-square test in Microsoft Excel. For all cases, *p*-values < 0.05 were considered significant.

## 5. Conclusions

In this study, we confirmed that 5-HT_1A_ or 5-HT_2C_ receptors are expressed in pMA of intramuscular origin. A dose-dependent serotonin stimulation resulted in decreased fat accumulation in the mature adipocytes, as well as decreased adipocyte proliferation in vitro. The microarray results displayed the patterns of transcriptome alteration, as well as key regulatory transcripts in the adipocytes during adipocyte differentiation from its precursor, preadipocytes, and after exogenous stimulation with serotonin and TNF-α. The transcriptomes alterations were associated with differentiation significantly involved with the enrichment of PPAR signaling pathway. Serotonin stimulation resulted in transcriptome alterations which were involved in activation of GPCR ligand-binding, cell chemotaxis, complement activation, and metabolic pathways, including metabolism of lipid, lipoprotein, and ketone bodies. Like serotonin, exogenous TNF-α stimulation also resulted in transcriptome modifications linked with proinflammatory responses in the porcine mature adipocytes of intramuscular origin.

## Data Availability

The MIMAE (minimum information about a microarray experiment) standard raw microarray dataset have been submitted to the NCBI-GEO database under the access number GSE141695.

## Supplementary Materials

Supplementary materials can be found at https://www.mdpi.com/1422-0067/21/2/638/s1.
