# Supplementary material for: Transcriptome Modifications in the Porcine Intramuscular Adipocytes during Differentiation and Exogenous Stimulation with TNF-α and Serotonin
Source: Int J Mol Sci. 2020 Jan 18;21(2):638. doi: 10.3390/ijms21020638 (PMC7013444; doi:10.3390/ijms21020638)
Supplement: Supplementary file 1 [file ijms-21-00638-s001.pdf]

Transcriptome Modifications in the Porcine Intramuscular Adipocytes during Differentiation and Exogenous Stimulation with TNF-α and Serotonin

Asuka Tada [1,2,†], Md Aminul Islam [1,2,3,†,‡], AKM Humayun Kober [1,2,†,‡], Kohtaro Fukuyama [1,2], Michihiro Takagi [1,2], Manami Igata [1,2], Leonardo Albarracin [1,4], Wakako Ikeda-Ohtsubo [1,2], Kenji Miyazawa [5], Kazutoyo Yoda [5], Fang He [5], Hideki Takahashi [6,7], Julio Villena [1,4], Hisashi Aso [2,8,*] and Haruki Kitazawa [1,2,*]

[1] Food and Feed Immunology Group, Laboratory of Animal Products Chemistry, Graduate School of Agricultural Science, Tohoku University, Sendai 981-8555, Japan; a-tada@morinagamilk.co.jp (A.T.); aminul.vmed@bau.edu.bd (M.A.I.); akmhumayuna@cvasu.ac.bd (A.K.M.H.K.); fukuyama.k.mc0511@gmail.com (K.F.); fukuyama.k.mc0511@gmail.com (M.T.); takagimichihiro@gmail.com (M.I.); lalbarracin@herrera.unt.edu.ar (L.A.); wakako.ohtsubo.a7@tohoku.ac.jp (W.I.-O.); jcvillena@cerela.org.ar (J.V.)

[2] Livestock Immunology Unit, International Education and Research Center for Food Agricultural Immunology (CFAI), Graduate School of Agricultural Science, Tohoku University, Sendai 981-8555, Japan

[3] Department of Medicine, Faculty of Veterinary Science, Bangladesh Agricultural University, Mymensingh 2202, Bangladesh

[4] Laboratory of Immunobiotechnology, Reference Centre for Lactobacilli, (CERELA-CONICET), Tucuman 4000, Argentina

[5] Technical Research Laboratory, Takanashi Milk Products Co., Ltd., Yokohama Kanagawa, Yokohama 241-0023, Japan; ke-miyazawa@takanashi-milk.co.jp (K.M.); k-yoda@takanashi-milk.co.jp (K.Y.); KA-HOU@takanashi-milk.co.jp (F.H.)

[6] Laboratory of Plant Pathology, Graduate School of Agricultural Science, Tohoku University, Sendai 981-8555, Japan; hideki.takahashi.d5@tohoku.ac.jp

[7] Plant Immunology Unit, International Education and Research Center for Food Agricultural Immunology (CFAI), Graduate School of Agricultural Science, Tohoku University, Sendai 981-8555, Japan

[8] Cell Biology Laboratory, Graduate School of Agricultural Science, Tohoku University, Sendai 981-8555, Japan

* Correspondence: asosan@tohoku.ac.jp (H.A.); haruki.kitazawa.c7@tohoku.ac.jp (H.K.); Tel.: +81-22-757-4311 (H.A.); +81-22-757-4373 (H.K.)

† These authors contributed equally to this work.

‡ Japan Society for the Promotion of Science (JSPS) Postdoctoral Fellow.

* **Corresponding authors:**

Dr. Haruki Kitazawa (haruki.kitazawa.c7@tohoku.ac.jp);

Dr. Hisashi Aso (asosan@tohoku.ac.jp)

Supplementary table

Table S1. Sequence information for the primers used for qRT-PCR-based quantification of mRNA expressions in porcine adipocytes after stimulation with serotonin and TNFα.

**Gene symbol**	**Primer sequence (3`- 5`)**	**Size (bp)**	**Accession No**
Beta actin	F: CAT CAC CAT CGG CAA CGA<br>R: GCG TAG AGG TCC TTC CTG ATG T	144	XM_003124280.5
INSR	F: AGA GCG GAT CGA GTT TCT CA<br>R: CCA TCC CAT CAG CAA TCT CT	245	XM_021083943.1
PPARγ	F: ACA CCG AGA TGC CGT T<br>R: CGA CAG GTC CAC AGA G	56	XM_005669788.3
PPARα	F: CGA CCT GGA AAG CCC GTT AT<br>R: GGA TCC ATC TGA TCC CGG AC	148	NM_001044526.1
TLR2	F: ACA TGA AGA TGA TGT GGG CC<br>R: TAG GAG TCC TGC TCA CTG TA	109	XM_005653577.3
TLR3	F: TAG AGA CAT GGA TTG CTC CC<br>R: AAC TTC TGG AAT GCA GGT CC	435	NM_001097444.1
TLR4	F: CTC TGC CTT CAC TAC AGA GA<br>R: CTG AGT CGT CTC CAG AAG AT	322	NM_001293316.1
EPCAM	F: GCGATA GCG ATT GTT GCT GG<br>R: CCC TAT GCA TCT CGC CCA TC	106	NM_214419.1
SAA2	F: AGAGCCTACTCGGACATGAGA GA<br>R: CCC CGG GCA TGG AAG TAC	65	NM_001044552.1
CXCL2	F: CCG GGA CCC CAC TGT GA<br>R: CAAACTTCCTGACCATTCTTGAGA	62	NM_001001861.2
CFB	F: CCT CGG GCT CCA TGA ATA TC<br>R: TGC CCC AAT GCT GTC TGA T	56	NM_001101824.1
C3	F: CCA ACA GGG AGT GCA ACG A<br>R: TGA CTC CGT GTC TGG GAC TTG	70	NM_214009.1
CSF1	F: CCA ACA GGG AGT GCA ACG A<br>R: TGA CTC CGT GTC TGG GAC TTG	147	NM_001244523.1
TGFB3	F: TTG CTA AAT GCT CCA GCC AG	90	NM_214198.1

	R: GCC TCC GCC TGT AGA ACA AG		
TNFAIP3 (A20)	F: CCC TGG GGC ATT ATG GGT TT<br>R: CCT CAC ACG TTG TAG CAC CT	60	NM_001267890.1
TNFα	F: CGA CTC AGT GCC GAG ATC AA<br>R: CCT GCC CAG ATT CAA AG	58	JF831365.1
IL-8 (CXCL8)	F: GCT CTC TGT GAG GCT GCA GTT<br>R: TTT ATG CAC TGG CAT CGA AGT T	62	NM_213867.1
IL6	F: TGG ATA AGC TGC AGT CAC AG<br>R: ATT ATC CGA ATG GCC CTC AG	109	NM_214399.1
IL1A	F: AGAATCTCAGAAACCCGACTGTTT<br>R: TTC AGC AAC ACG GGT TCG T	62	NM_214029.1
IL1B	F: GCC CTG TAC CCC AAC TGG TA<br>R: CCA GGA AGA CGG GCT TTT G	61	NM_001302388.2
CYP1A1	F: GCC CGA CCT CTA CAG CTT<br>R: TGG GAT GGT GAA GGG GA	843	NM_214412.1
FABP4	F: TCA CTG CAG ATG ACA GGA AAG TC<br>R: ACC AGG GCG CCT CCA T	58	NM_001002817.1
CD36	F: AAA ATA TAA CCC AGG ACC CTG AGA<br>R: ATG GCA CCA TTG GGC TGT A	61	NM_001044622.1
LPIN1	F: AAA ACC TGT CCC CAG TCG TG<br>R: GAA ACT GAA GGA CTG GCG CT	91	NM_001130734.1
PLIN2	F: CTG ACC GAG TCT CAG GAT GC<br>R: GCT GGC AGA GGC AGG TTT A	102	NM_214200.2
ACAA1	F: TGT CCA TGT GTA TCG GGA CC<br>R: CAC CAT CGG TGC TAG AGG TG	147	XM_003132103.4
GLP2R	F: CTG TAC ACG GTG GGC TAC TC<br>R: CGA AGA CAC AAG AGG AGG GTC	70	NM_001246266.1
HTR1F	F: TGG CTT GGA TAT CTC AAT TCC CT<br>R: TCG ACA TCG TAC AAG TTT TTG G	96	NM_214101.1
HTR2A	F: GGT CGG CCT TTT CCA GGT AT<br>R: GGC CTT GTC GTC TTG CTT TG	152	NM_214217.1

KLF15	F: CCT GTG AAG GGG GAG CAT TT<br>R: ACC CCT GGC TCC ATG TTT TC	128	NM_001134349.2
FGFR1	F: GAC TCT CCA GAG GCG GAA TC<br>R: TGC GGT TAG AGG TTG GTG AC	115	XM_005671767.3
CADM4	F: CGA AAG GAG CTG AAA GGG GT<br>R: TGC CTC GCA GAT GAC GAT AC	120	XM_021094480.1
AMCF2	F: AGT GCT CCA AGG CAG AAG TGA<br>R: TTT GGG TCC AGA CAG ACT TCC T	64	NM_213876.1
THBD	F: AGG CTT GCG AGC ATC TAC AG<br>R: GGT TGT CCC CTG TAA CCC AC	192	NM_001130732.1
PROC	F: GAG CCA AAG GTG AGG TCC C<br>R: ATG GGG ACT GCT TCC CAT TG	142	NM_213918.1
NOTCH4	F: CCA GGC TGA AGA AAT GGC CT<br>R: GTC ACT CCA TCA GGT CCA CG	153	NM_001123147.1
NKRF	F: CCA AAT CGT ACA TGG CGC TG<br>R: CCT GGG TGA AGA GCC TGT TT	180	XM_001927183.6
PTGS1	F: AGC TCA AGT TTG ACC CGG AG<br>R: CGA CCC CAT AGT CCA TCA GC	186	XM_001926129.6
PTGIS	F: CCG ATT CCT GAA CCC GGA TG<br>R: CCC AGG GCA GGC TGT AAT TC	83	XM_005654248.3
PTGDS	F: GTC CAT GTG CAA ATC GCT GG<br>R: GCA TCA GAG TTC GGG TCA CA	101	NM_214228.1
ALOX12	F: ACC ACC AAG GAA GAC GTG AC<br>R: GGC CTT GGG GCT TGA GAA AT	162	XM_003483092.4
NOS2	F: CAG CCA GGT GCT CAC CTA TT<br>R: TCT CAA GCC TCT GCC TTT CG	110	NM_001143690.1
